# Cell surface proteins in *S. pneumoniae*, *S. mitis* and *S. oralis*

**Published:** 2011-06

**Authors:** A Madhour, P Maurer, R Hakenbeck

**Affiliations:** Department of Microbiology, University of Kaiserslautern, Paul-Ehrlich Str. 23, D-67663 Kaiserslautern

**Keywords:** *Streptococcus pneumoniae*, *Streptococcus mitis*, *Streptococcus oralis*, cell surface protein, virulence factors, choline binding proteins

## Abstract

**Background and objectives:**

*Streptococcus pneumoniae*, a major human pathogen, is closely related to the commensal species *S. mitis* and *S. oralis*. *S. pneumoniae* surface proteins are implicated in virulence and host interaction of this species, but many of them have recently been detected in *S. mitis* B6 *in silico*. We tested for the presence of such genes usinga set of eight *S. mitis* and eleven *S. oralis* strains from different geographic locations.

**Materials and Methods:**

An oligonucleotide microarray was designed based on the genomes of *S. pneumoniae* R6 and TIGR4 as well as *S. mitis* B6 to include 63 cell surface proteins. The *S. pneumoniae* genes encoding neuraminidases, hyaluronidase and pneumolysin were also included. In addition to comparative genomic hybridization experiments, homologues were identified *in silico* in the genome of *S. oralis* Uo5.

**Results and Conclusions:**

The results document that many *S. pneumoniae* related surface proteins are ubiquitously present among the Mitis group of streptococci. All 19 samples hybridized with the *pavA* probe representing a gene important for adherence and invasion of *S. pneumoniae*. Only eight genes were not recognized in any strain, including the *S. pneumoniae* PcpC gene as the only virulence gene of the *S. pneumoniae* core genome.The fact that only 12 out of 26 genes present in the *S. oralis* Uo5 genome could be detected by microarray analysis confirms the sequence variation of surface components.

## INTRODUCTION

*Streptococcus pneumoniae* colonizes the nasopharynx of humans early in life. It is considered as one of the major human pathogens, causing a variety of invasive diseases including meningitis, pneumonia and septicemia. Disease occurs frequently in children and in people with deficiencies in the immune system or is associated with damage to the mucosal surface (e.g. by viral infections). In contrast, the closest relatives, *S. mitis* and *S. oralis*, are commensal bacteria that reside in the upper respiratory tract and the oral cavity. They rarely do cause disease such as endocarditis especially in immunocompromised patients (1–4).

The reason for the large difference in virulence potential among these members of the Mitis group is not fully understood. Many gene products have been described as *S. pneumoniae* virulence factors on the basis of mouse infection models. However, most of them are present in the first completed *S. mitis* genome of strain B6 (5). Thus only a few are apparently pneumococcal specific components, including the capsular cluster, genes encoding surface proteins such as the choline binding proteins PspC (CbpA), PspA and PcpA, the hyaluronidase HlyA and a genomic island that contains *ply* plus *lytA* encoding the potent cytolysin pneumolysin and the major autolysin. The genes *ply* and *lytA* have been identified in several *S. mitis* (5–8). The LytA gene frequently is part of prophages (9).

Members of the Mitis group are naturally transformable, documented by the presence of mosaic genes as a result of interspecies gene transfer and recombination events. A paradigm for this scenario are penicillin-binding proteins, the target proteins for beta-lactam antibiotics (10–13). Moreover, recombinations between *S. pneumoniae* genes and other members of the Mitis group have been observed for the virulence genes encoding neuraminidase A and IgA protease (14, 15). As a consequence, *S. pneumoniae* contains a large accessory genome. Genomes of *S. pneumoniae* clones vary by over 10%, and probably less than 50% of all genes are common to all *S. pneumoniae* strains (16, 17). Similarly, the accessory genome of *S. mitis* B6 has been estimated to constitute over 40% of all coding sequences (5).

Cell surface proteins play a central role in the interaction with host cells, and many of them are listed as pneumococcal virulence factors (18, 19). Choline-binding proteins (CBPs) represent one large group of such proteins. They are anchored to the cell wall by hydrophobic interactions with choline-containing teichoic acids [for review, see (20)]. They are composed of a choline-binding module consisting of repeats of 20 amino acids and a nonconserved functional domain. Some CBPs are considered to be important virulence factors specific for *S. pneumoniae*, such as PcpA, PspA and PspC. On the other hand, *S. mitis* B6 contains a large number of CBPs, many of which have unusual repeat domains with a 40mer repeat motif and are part of the accessory genome (5).

Another group of cell wall surface proteins of Gram-positive bacteria are covalently linked to the muropeptide of the peptidoglycan layer, and are characterized by an LPXTG motif localized mainly at the C-terminal end (21). Members of this family of cell surface anchor proteins (LPXTG proteins) exhibit protease or glycosylase activity of various specificities, or represent adhesion molecules to ensure close contact to host cells; the function of many of them is still unknown. Several LPXTG proteins whose presence has been linked to *S. pneumoniae* pathogenicity are part of the accessory genome, such a large serine-rich protein and associated proteins responsible for glycosylation and export (22). This genomic islet occurs also in *S. gordonii* where the protein named GspB has been associated with endocarditis (23). It has also been detected in the *S. mitis* genome (5). This shows that not only antibiotic resistance genes are part of the gene pool common to all members of the Mitis group, but components that might contribute to the modulation of the pathogenicity are candidates as well.

Understanding the distribution of cell surface components among members of the Mitis group will contribute to our understanding concerning the evolution of these species. Moreover, the presence of surface proteins homologous to *S. pneumoniae* components in related species has important implications for the design of protein-based pneumococcal vaccines.Therefore, an oligonuc-leotide microarray was designed to cover genes encoding surface proteins based on the genomes of *S. pneumoniae* R6 and TIGR4 as well as *S. mitis* B6. Comparative genomic hybridizations were performed using nineteen *S. mitis* and *S. oralis* strains from different geographic areas, all of which have been typed by multi locus sequence typing [MLST; (24)] to ensure correct speciation (13). The *S. oralis* Uo5 genome which is the only finished genome of this species (25) was searched for homologues of the genes represented on the microarray to estimate the efficiency of the hybridization approach.

## MATERIALS AND METHODS

**Bacterial strains**. Strains used in the present study are listed in Table 1. All were typed by MLST analysis using the primers as specified by Chi et al. (13).

**Table 1:** Streptococcus spp. strains.

Strain	origin/properties1	year of isolation	Pen resistance	reference
*S. pneumoniae*				
R6 (ATCC BAA-255)	rough 2	1944	S	(31)
*S. mitis*				
B6	GER	1994	R	(26)
NCTC10712	UK	1967	S	(32)
RSA37	SA	1986/87	R	(13)
Uo1	HUN	<1992	R	(5)
RSA04	SA	1986/87	R	(13)
M3	SA	1986/87	S	(16)
S492	SP	1993	R	(13)
SV10	SP	1992	S	(13)
SV5	SP	1992	S	(13)
*S. oralis*				
RSA11	SA	1986/87	R	(13)
RSA18	SA	1986/87	R	(13)
RSA20	SA	1986/87	S	(13)
RSA40	SA	1986/87	R	(13)
Uo2	HUN	<1992	R	(13)
Uo3	HUN	<1992	R	(13)
Uo5	HUN	<1992	R	(11)
Uo17	HUN	<1992	R	(13)
S510	SP	1993	R	(13)
S527	SP	1993	R	(13)
S767	SP	1993	R	(13)

^1^ GER: Germany; UK: United Kingdom; SA: South Africa; HUN: Hungary; SP: Spain. ^2^ *S. pneumoniae* R6 is an unencapsulated derivative of the type 2 strain D39 (32).

**Comparative genome hybridizations and data analysis**. The 70-mer oligonucleotide microarrays representing genes of *S. pneumoniae* R6 and TIGR4, and *S. mitis* B6, have been described (5). Oligonucleotides representing cell surface protein genes covered all choline-binding protein and LPXTG cell wall anchor protein genes found only in *S. mitis* B6 as well as those present specifically in *S. pneumoniae* R6 and TIGR4 but not in *S. mitis* B6. In case of homologues being present in *S. mitis* and *S. pneumoniae*, two oligonucleotides specific for each homologue were included. In general, the oligonucleotides were designed to match the non-repeat regions of LPXTG protein genes and CBP genes in order to avoid crosshybridization. In addition, four new 70 mer oligonucleotides representing variable sequences of the following genes were included: spr 0351-2 GCTACGA ATACCAACAAACATCATGGGGAAGAATATGAT AGCCAAGCAGAGAAACGAGTCTATTATTTTG; smi_0934 (*pce1*, choline-binding protein E, *lytD1*) GGCTCAAAGAACGAGGAATTGAGAGAAT-CAACGCAGCCAGCAAAGACTATGATGCAA-CAGTTTTTGATAT; smi_0091-2 (cell wall surface anchor family protein) GCCTGCTGACACCA-TGACAAGCTCTACCA ATACG ATGGCAG-GTGAAAACATGGCTGCTTCTGCTAACAAG and smi_1662-2 (*mon*X, cell wall surface anchor family protein, Ser rich) GGATCTGTGTTACT-TGGAGCTCTAGCAGCTGTTACAGGTATTG-G AT T G G T T G C G A A A C G T C G TA A G C G G G. Oligonucleotides (30 pmol/µl) were spotted on Nexterion HiSens Slides E (SCHOTT Jenaer Glas GmbH) using the SpotArray TM24 Microarray SpottingSystem (Perkin Elmer) with 32 SMP3-Pins (Telechem).

**DNA labelling and hybridization**. Chromosomal DNA was isolated as previously described (5). 5 µg of heat denatured genomic DNA was used as a template for direct incorporation of alternate fluorescent analogues Cy5-and Cy3-dCTP (Perkin Elmer, Boston, USA) by randomly primed polymerization reaction. Ethanol precipitated labeled DNA was resuspended in hybridization buffer (Nexterion Hyb, Formamid 1:1) and denatured twice at 95°C for 5 min. Hybridization was performed following the manufacturers’ recommendations using a hybridization temperature of 40°C for 16 h. Labeled chromosomal DNA of *S. mitis* B6 was used as reference. Data Processing Microarrays were scanned on a laser scanner (ScanArray 4000 Microarray Analysis System, Perkin Elmer Life Sciences) with alow resolution of 50 µm using Scan Array Express Software,Version 2.1. Photomultiplier Tube (PMT) was adjusted to balance the two fluorescence channels and biochips were scanned with a 10 µm resolution. Replicate spots that had only background values as estimated from the negative controls included on themicroarray were discarded. For each experiment, the fluorescence intensity of the test strain was normalized to that obtained for the B6 reference. Signals that showed an intensity ratio of 0.3 and above were considered to be positive.

**Accession numbers**. Oligonucleotide microarray: oligonucleotides used in the present study are listed under the ArrayExpress accession numbers A-MEXP-1772 and A-MEXP-1755. The pathochip is accessible under Array Express accessiou No. E_MEXP_3360

Accession numbers of genomes cited in the text: *S. pneumoniae* R6 GenBank accession number AE007317.1; *S. pneumoniae* TIGR4 GenBank accession number AE005672.3; *S. mitis* B6 EMBL accession number FN568063; *S. oralis* Uo5 EMBL accession number FR720602.

## RESULTS

**Fig. 1:**
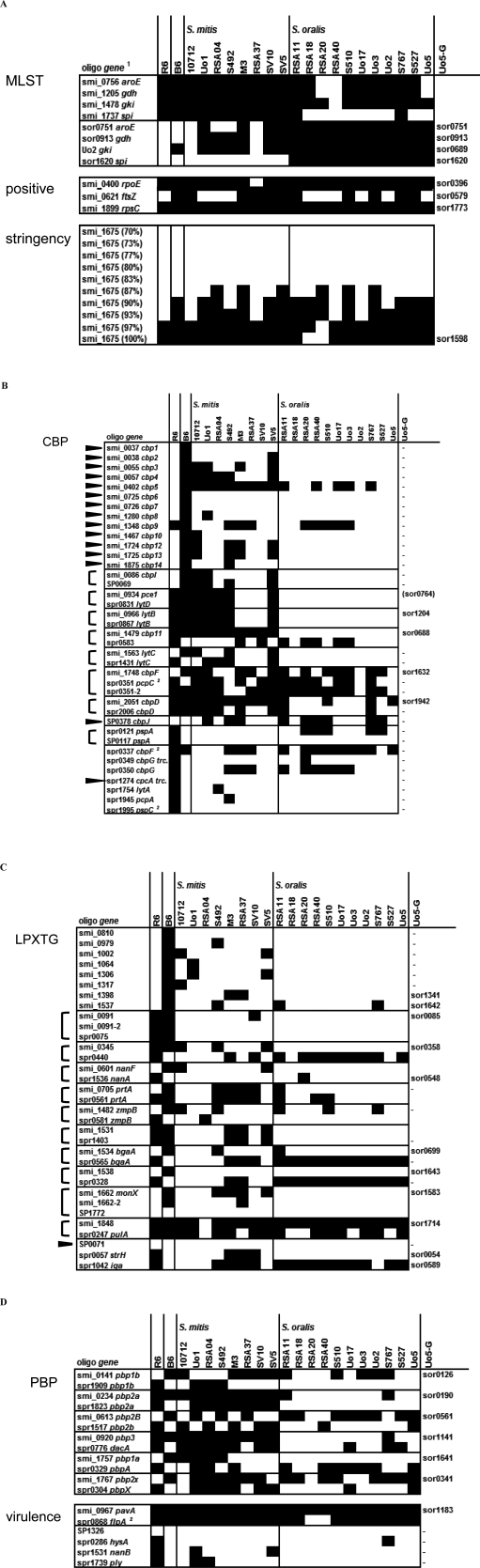
Genomic hybridization signals in pairwise comparisons between *S. mitis* B6 and *S. mitis* and *S. oralis* strains. *S. pneumoniae* R6 DNA was also included. Black signals indicate positive hybridization signals.^1^ The gene numbers and names are those of the published genome sequences; hypothetical and conserved hypothetical genes are not specififed. ^*2*^ *S. pneumoniae* R6 genes whose name differs from that of TIGR4. Features representing genes specific for *S. mitis* B6, or *S. pneumoniae* R6 genes that are absent in *S. pneumoniae* TIGR4 and vice versa, are indicated by black arrow. Oligonucleotides that represent homologues of different strains with variable sequences are indicated by brackets. Genes present in the *S. oralis* Uo5 genome (Uo5-G) are indicated on the right; absence of genes is marked by.

**The oligonucleotide microarray and control features**. The microarray includes two negative controls (random oligonucleotides), stringency controls with a stepwise decrease of homology from 100% to 70% to a *S. mitis* B6 gene, as well as oligonucleotides representing genes used for MLST analysis which should allow differentiation between *S. mitis* and *S. oralis*. No signal with any of the strains was obtained with the negative controls. *S. mitis* B6 DNA hybridized with the stringency features of 90% homology and above (Fig. 1A). All *S. mitis* strains hybridized with the 97% and 100% stringency oligonucleotides, as did nine of the eleven *S. oralis* strains, documenting variation in gene sequences in some *S. oralis* (RSA18 and RSA20). The *S. mitis* specific MLST genes hybridized with all *S. mitis*, and those specific for *S. oralis* with all *S. oralis* strains, confirming the species specificity for these features. Nevertheless, only with the *spi* gene encoding the signal peptidase I, unambiguous results were obtained (i.e. only hybridization with the matching species was detected).

Three oligonucleotides specific for *S. mitis* B6 represented genes that are present in all eubacteria encoding the ribosomal protein S3 (*rpsC*), the delta-subunit of RNA polymerase (*rpoE*), and FtsZ required for cell septation. Only with *rpsC* signals were obtained with all strains, suggesting a high degree of conservation of this gene within the Mitis group, and sequence variation in the other genes.

With *S. pneumoniae* R6 DNA, all spr-based oligonucleotides were recognized; similarly, with *S. mitis* B6 DNA signals were obtained for all smi-based oligonucleotides. Oligonucleotides specific for genes that are present in *S. pneumoniae* TIGR4 but absent in both, *S. pneumoniae* R6 and *S. mitis* B6, were not detected with DNA of these two strains.

**Choline-binding proteins**. Altogether a total of 29 CPB genes are represented on the microarray. Homologues of seven genes occur in both, *S. pneumoniae* and *S. mitis* B6, including the physiologically important genes *lytB, cbpF* and *cbpD*. These genes are represented with at least two primers each matching the sequences of the two species. Furthermore, the microarray contains 13 CBP genes of *S. mitis* B6 that are absent in *S. pneumoniae*, and eight genes present in *S. pneumoniae* R6 and/or TIGR4 which are not found in *S. mitis* B6. It is somewhat confusing that the annotation of CBPs in *S. pneumoniae* R6 differs from that in the TIGR4 strain: *spr0351* is named *pcpC* but is the CbpF gene, and *spr0337* named *cbpF* corresponds to *cbpC*, a paralogue of *cbpF*. Moreover, the genes spr0349 and spr0350 are fragments of the gene SP0390 (*cbpG*).

In general, with *S. mitis* DNA more positive hybridization signals were obtained than with *S. oralis* DNA (Fig. 1B). Twenty-two genes were recognized by at least one *S. mitis*, and ten reacted with at least one *S. oralis*. Positive signals of a single strains varied between one (*S. oralis* Uo2) to 20 (*S. mitis* SV5). Not one oligonucleotide hybridized with all strains, but the two genes *cbpF* and *cbpD* reacted with the DNA of most *S. mitis* and *S. oralis* strains. Three features were recognized by all *S. mitis* strains representing *S. mitis cbp5*, *cbp11*, and *cbpD*. The three *S. mitis* B6 specific CBPs could not be detected in any strain, as were *S. pneumoniae pspC* and *spr1274*, a gene fragment not present in the TIGR4 strain. It should also be noted that the *lytA* feature was designed to be specific for the *S. pneumoniae*gene. Only one of the three *S. mitis* strains known to harbour a *lytA* homologue (5) hybridized with this gene (RSA04) whereas *S. mitis* B6 and Uo1 did not, indicating sequence variation in the latter two strains.

**LPXTG cell wall anchor proteins.** The microarray contains 33 oligonucleotides representing 21 genes encoding cell wall surface anchor proteins (LPXTG proteins). Ten of them occur in *S. mitis* B6 and *S. pneumoniae*. Eight are found only in *S. mitis* B6 and not in *S. pneumoniae*, and three occur in *S. pneumoniae* R6 and/or TIGR4 but not in *S. mitis* B6 as indicated in Fig. 1C. The oligonucleotides of the microarray match non-repeat sequences in order to detect specifically the presence of regions representing the functional domain. None of the strains reacted with all genes, and not one hybridized with the *S. pneumoniae* TIGR4 gene *SP0071* encoding the zinc metalloprotease ZmpC (Fig. 1C). As with CBPs, more genes were recognized with *S. mitis* DNA (19 genes were detected in at least one strain) compared to *S. oralis* where nine genes were detected. Most *S. mitis* B6 specific LPXTG protein genes were not recognized with *S. oralis* DNA except *smi_1537* encoding a putative N-acetyl-beta-hexosaminidase.

Three strains hybridized with the neuraminidase gene *nanA* or the homologue *nanF* of *S. mitis* B6 (*S. mitis* 10712 and SV5, and *S. oralis* RSA20). All *S. oralis* contained sequences of *bgaA*, *pulA*, *spr0328* (the *smi_1538* homologue) encoding a beta-galactosidase, pullulanase, and a protein of unknown function, respectively. The huge serine-rich protein *S. mitis monX* (*smi_1662*) was detected in some *S. mitis* strains. Most strains contained an IgaA protease gene *spr1042* which is absent in *S. mitis* B6.

**Penicillin-binding proteins** Oligonucleotides specific for penicillin sensitive *S. pneumoniae* and the high-level resistant *S. mitis* B6 were used for each of the six PBPs. *S. mitis* B6 PBPs are highly divergent from the *S. pneumoniae* sequences (26), and thus B6 DNA reacted highly specifically only with B6 oligonucleotides (Fig. 1D). A variable pattern of hybridization signals within both *S. mitis* and *S. oralis* was observed for *pbp2x* and *pbp2b*. Interestingly, many *S. oralis* strains hybridized with the *pbp1a* sequence specific for *S. pneumoniae*. The features representing *pbp2a* and *pbp3* discriminated largely between the two species *S. oralis* and *S. mitis*.

***S. pneumoniae* specific virulence genes** Five genes implicated in *S. pneumoniae* virulence were chosen in the present study, four of which are absent in *S. mitis* B6: two genes present in all *S. pneumoniae* strains *ply* (spr1739) and *hysA* (*hylA; spr0286)*, *nanB* (*spr1531*) and *nanC* (*SP1326*) variably present in *S. pneumoniae* (Fig. 1D). PavA (FlpA in R6) has been implicated in adherence and invasion of *S. pneumoniae*, but is present in *S. mitis* B6 and thus cannot be considered to be an *S. pneumoniae* specific virulence factor (5).

All strains hybridized with *pavA*/*flpA*. SP1326 encoding a neuraminidase gave only negative signals, and *hysA*, *nanB* and *ply* hybridized with only one or two strains. Only two strains hybridized with *ply*: *S. mitis* Uo1 and RSA04 (Fig. 1D).

**Genes in the *S. oralis* uo5 genome**. It is obvious that due to sequence variation, the absence of a hybridization signal does not necessarily indicate absence of a gene. Therefore, the genes represented on the microarray were searched in the genome of *S. oralis* Uo5 (Fig. 1A-D, last lane Uo5-G). Considering the 61 genes encoding LPXTG proteins, CBPs, PBPs and *S. pneumoniae* virulence factors, only 22 were present in the *S. oralis* Uo5 genome, but only 10 were detected by hybridization to the microarray. One false positive signal was obtained with *spr033*7, a paralogue of the CbpF gene *spr0351*. The CbpF gene is present in *S. oralis* Uo5, therefore, the signal is likely to be due to sequence similarity between these two genes.

## DISCUSSION

The present study clearly documents that homo-logues to *S. pneumoniae* surface protein genes are not only common in *S. mitis*, but that many of them are widespread also among *S. oralis*. The strains used here covered different geographic areas and distinct MLST profiles in order to ensure a wide variety of genotypes. In a recent report using a microarray representing *S. pneumoniae* virulence genes, only five *S. mitis* and one *S. oralis* were used. In this case, the single *S. oralis* strain probably was not representative for this species since greater hybridization signal was obtained compared to the four *S. mitis* strains (27).

**Fig. 2:**
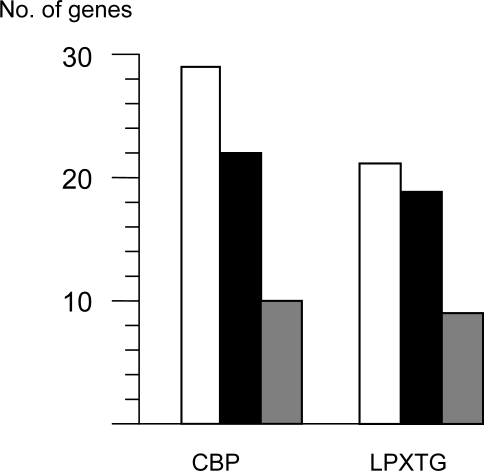
CBP and LPXTG protein genes present in *S. oralis* and *S. mitis* strains as indicated by hybridization signals. White: total number of genes on the microarray (homo-logues were calculated as one gene), positive signals in at least one *S. mitis* (black) and in at least one *S. oralis* (grey).

The data confirm the close genetic relatedness between *S. mitis* and *S. pneumoniae*. Not considering the control oligonucleotides, 50 out of the 61 genes represented on the microarray hybridized with at least one *S. mitis* strain, whereas only 27 genes were recognized among *S. oralis* (see Fig. 2 for LXPTG protein and CBP genes). In this context, it is important to consider that the data represent a minimal number, since negative results are obtained not only in the absence of a gene but also in case of highly variable sequences, and variability is frequent especially in cell surface components. This became evident in case of *S. oralis* Uo5 where only ten genes of the 22 surface protein genes present in the genome were detected by the microarray analysis. Many of the surface proteins appear to be part of the accessory genome, i.e. are also variably present in *S. pneumoniae* genomes or in *S. mitis* B6 (5). More genomic data will be required to be able to estimate the core surface proteins of the three streptococcal species.

Common to most *S. mitis* and *S. oralis* strains were *cbpD* involved in hydrolysis of the peptidoglycan and interestingly also *cbpF*. CbpF has been implicated in control of another CBP, the murein hydrolase LytC (28). However, *lytC* signals were not detected in any of the *S. oralis* strains, nor is a *lytC* homologue present in *S. oralis* Uo5 genome, suggesting other functions of CbpF. CBPs appear to be common also in *S. oralis*, which indicates the presence of choline-containing teichoic acids as well. The *lic* locus involved in teichoic acid biosynthesis is present in a modified version in *S. oralis*, suggesting that teichoic acids might have a structure distinct from that of *S. pneumoniae* (29). In this context, it is noteworthy that a CBP of unknown function which contains a 40mer repeat motif and which is highly conserved in *S. pneumoniae* and *S. mitis* B6 (*smi_1479* respectively *spr0583*) also appears to be present in *S. oralis*. LytB was not detectable in any strain but is essential for cell septation, documenting a high degree of sequence variation as apparent from the *lytB* sequences of *S. mitis* B6 and *S. oralis* Uo5. Among the *S. pneumoniae* genes not detected in any of the strains was *pspC* (*cbpA, hic*), which is located on a *S. pneumoniae* specific region together with the two component system TCS06. However, *pspA* and *pcpA* which are absent in *S. mitis* B6 and are considered to be *S. pneumoniae* specific surface proteins were detected in several *S. oralis* respectively in one *S. mitis*. Since CBPs evolve rapidly via duplication and recombination as exemplified in the *S. mitis* B6 genome (5), DNA sequence analysis will be required to confirm this observation on the molecular level.

A similar picture is seen concerning the LPXTG cell surface protein genes. There are several features where *S. oralis* strains gave positive results, including the beta-galactosidase gene *bgaA*, the pullulanase gene *pulA*, the neuraminidase *nanA* and at least one the zinc metalloproteases *zmpB* and *igA*. The *nanA* sequences of oral streptococci cluster closely together, and this has been interpreted as an indication of frequent genetic exchange at this locus (27), as documented between *S. pneumoniae* and *S. oralis nanA* (14). The Ser-rich LPXTG gene *monX* (*psrP*) was identified in some *S. mitis* in the present analysis, whereas it was not found in the strains studied by Johnston et al. (27). It is obvious that the sequence variation is high, and thus also no signal was detected for *monX* with *S. oralis* Uo5 DNA although the gene is present in its genome sequence.

The high variability of signals obtained with *pbp* sequences, especially *pbp2x*, *pbp2b* and *pbp1a* involved in penicillin resistance, was expected, since most strains were resistant to beta-lactams and thus are likely to contain mosaic *pbp* genes (13). On the other hand, the *pbp2a* oligonucleotides hybridized with *S. mitis* DNA, and not one *S. oralis* hybridized with the *S. pneumoniae* sequence, similar to results obtained with an Affymetrix microarray based on the *S. pneumoniae* TIGR4 genome (6).

Typical virulence genes were absent in the vast majority of *S. mitis* and *S. oralis* strains with the exception of *pavA*, confirming the widespread presence of this gene and the importance of PavA for adherence also in other oral streptococci.The only two strains hybridizing with the pneumolysin gene *ply*, *S. mitis* Uo1 and RSA04, also contain a LytA gene which is probably part of a prophage (5). In *S. pneumoniae, ply* and *lytA* are located together on a small pathogenicity island (5), and thus the localization of *ply* in the two *S. mitis* strains is important in the context of evolution of *S. pneumoniae*. Preliminary data suggest that at least in *S. mitis* Uo1, a similar island is present (unpublished results). Surprising was the detection of the hyaluronidase gene *hlyA* in one strain, *S. oralis* S767, since hyaluronidase activity had not been detected among oral streptococci so far (30). It would be interesting to determine whether this strain indeed contains a functional enzyme.

In conclusion, the data reveal that many cell surface proteins are common to the three species *S. pneumoniae*, *S. mitis* and *S. oralis*. The term ‘virulence factor’ used for several of these proteins for *S. pneumoniae* is thus questionable. It is obvious that proteins implicated in adhesion and attachment to host cells must be present in commensal species as exemplified by *pavA*, and thus should be considered as factors essential for host interaction independent on the pathogenicity potential of the bacteria.
